# Association Between Serum Lactate and Unsatisfactory Outcomes in Critically Ill Children in the Immediate Post-operative Period of Liver Transplantation

**DOI:** 10.3389/fped.2021.796504

**Published:** 2022-01-24

**Authors:** Jaime Fernández-Sarmiento, María Angélica Wilches-Cuadros, Ricardo Hernandez-Sarmiento, Hernando Mulett, Karen Moreno-Medina, Nicolás Molano, Julián Augusto Palomar Dominguez, Lorena Acevedo, Claudia Salinas, Jairo Rivera

**Affiliations:** ^1^Department of Pediatrics and Intensive Care, Fundación Cardioinfantil-Instituto de Cardiología, Universidad de La Sabana, Bogotá, Colombia; ^2^Department of Pediatrics and Intensive Care, Fundación Cardioinfantil-Instituto de Cardiología, Universidad el Rosario, Bogotá, Colombia; ^3^Research Department, Fundación CardioInfantil-Instituto de Cardiología, Bogotá, Colombia; ^4^Clinical Research Group, School of Medicine and Health Sciences, Universidad del Rosario, Bogotá, Colombia; ^5^Department of Pediatrics, Universidad de La Sabana, Bogotá, Colombia; ^6^Department of Liver Transplantation, Fundación Cardioinfantil-Instituto de Cardiología, Bogotá, Colombia

**Keywords:** biliary atresia, child, hypoxia, liver failure, mortality, tissue donor

## Abstract

**Objectives:**

Serum lactate is a useful biomarker of tissue perfusion in critically ill patients. We evaluated the behavior of serum lactate in children in the pediatric intensive care unit (PICU) immediately after liver transplantation and its association with surgical complications, graft dysfunction and 90-day mortality.

**Materials and Methods:**

A prospective observational study carried out between November 2009 and December 2019. Multidisciplinary PICU at the University Children's Hospital, Fundación Cardioinfantil-IC, Bogotá, Colombia.

**Measurements and Main Results:**

Patients between 1 month and 18 years of age who were in the immediate post-operative period following living-donor or cadaveric liver transplantation were included. A total of 145 patients with a median age of 14 months (IQR 8–60) met the inclusion criteria. Biliary atresia was the main diagnosis in 56.5% of the cases. A serum lactate level > 3.0 mmol/L on admission to the PICU was associated with biliary complications (AUC 0.73 95% CI 0.54–0.93; *p* = 0.05) and mortality (AUC 0.72 95% CI 0.63–0.8; *p* = 0.01). A lactate level > 2 mmol/L after 6 h in the PICU was associated with mortality (AUC 0.70 95% CI 0.54–0.83; *p* = 0.02). Higher lactate levels and lack of clearance were associated with the presence of *tardus et parvus* waveforms (*p* = 0.001) on liver Doppler, primary dysfunction (*p* < 0.001), arterial thrombosis (*p* < 0.001) and neurological complications (*p* = 0.04). There was an inverse correlation between admission lactate and the volume of fluids administered during surgery (rho = 0.36; *p* < 0.001). A total procedure time > 350 min, along with a vasopressor score > 7 and elevated lactate, were associated with worse outcomes (*p* < 0.001).

**Conclusions:**

In post-operative pediatric liver transplant patients, the level of serum lactate is associated with post-operative surgical complications and mortality.

## Introduction

Acute and chronic liver failure in childhood has an almost 90% mortality rate without transplantation (1, 2). Liver transplantation has developed significantly over the last 40 years and is the definitive solution for various liver diseases which lead to cirrhosis (3). Biliary atresia is the most common cholestatic disease in children and is responsible for ~40% of liver transplant cases in children (2, 4, 5).

Complications of liver transplantation are frequent and, due to the complexity of the procedure, are largely determined by each center's level of experience. The SPLIT registry, over a period of seven years in 39 North American centers, found that the main complications in the first post-operative month were the need for reintervention (31.7%), hepatic artery thrombosis (6.3%), and portal vein thrombosis (3.2%) (6–8). In the first 90 days, biliary tract complications were reported in 13.6%, and in the first year, acute cellular rejection occurred in 34.7% (1).

Tissue perfusion disorders in critically ill children are often evaluated by measuring serum lactate. In post-operative patients and those with sepsis or multiple organ failure, serum lactate is associated with microcirculatory dysfunction, worse outcomes and mortality (9). Under normal conditions, lactate is eliminated by enzyme processes carried out in the liver, muscles and kidneys. Although serum lactate is expected to be elevated in the anhepatic phase of liver transplantation, it is often used in the perioperative and post-operative periods in the pediatric intensive care unit (PICU) to evaluate tissue perfusion and possible associated complications (10, 11).

In this regard, given that the levels found in the blood are a product of the relationship between production and clearance, recent studies in adults have found an association between lactate clearance and early graft dysfunction and death (12). We are not aware of any studies in children evaluating the behavior of serum lactate in post-operative living-donor or cadaveric liver transplant patients. The aim of this study was to evaluate the behavior of serum lactate levels following liver transplantation and their association with surgical and medical complications, the need for vasopressor support and death.

## Materials and Methods

### Study Design and Context

This was a prospective, analytical observational study of children immediately after liver transplantation between November 2009 and December 2019 at the Fundación Cardioinfantil in Bogotá, Colombia, which is a reference center for liver transplantation in children and adults. Each patient's demographic data, clinical characteristics and comorbidities were taken from the service's database. Likewise, the variables of interest for the study were recorded prospectively. The information was completed using the electronic clinical chart. Data on the first 48 h after transplantation were taken, with the first 90 days after transplantation used to evaluate mortality. This study was approved by the institutional research and ethics committees, with approval number CEIC-3996-2019.

### Participants

All patients between 1 month and 18 years of age who were in the immediate post-operative period following living-donor or cadaveric liver transplantation were included. Patients who concomitantly received another organ besides the liver (simultaneous liver-kidney or heart), those who experienced cardiopulmonary arrest during the surgical procedure, patients with other primary causes of hyperlactatemia (inborn errors of metabolism, diabetes mellitus, neoplasms or the use of beta adrenergic prior to transplant) and children with intestinal malabsorption were excluded.

### Variables

Demographic and clinical data were analyzed, and ultrasound and Doppler were performed in the immediate post-operative period within 48 h of surgery. The resistive index (a hepatic artery flow wave calculation: the difference between maximum systolic velocity and end-diastolic velocity over maximum systolic velocity), was considered to be abnormal when >0.75 or if *parvus et tardus* waveforms (an ultrasound pattern resulting from arterial stenosis, due to reduced blood flow through the narrowed vessel) were present. If a complication was detected on ultrasound, it was confirmed by tomography. Biliary complications such as fistulas or biliary stenosis were confirmed intraoperatively or with biliary scintigraphy.

The primary outcome was the post-operative serum lactate level associated with surgical complications and primary graft dysfunction. The secondary outcomes were the serum lactate levels associated with length of stay in pediatric critical care, days of mechanical ventilation, need for vasopressor support and death.

### Data Source and Definitions

Serum lactate was collected from the arterial gases recorded in the electronic clinical chart and corroborated with the clinical laboratory records. Lactate was measured 0–1 h after post-operative admission [Time 0 (T0)], between 1 and 6 h [Time 1 (T1)], between 6 and 24 h [Time 2 (T2)], and between 24 and 48 h [Time 3 (T3)] after surgery. The percentage of lactate clearance was defined using the following formula: lactate at hour 0 of admission to the intensive care unit minus lactate at hour 6, divided by lactate at hour 0 and multiplied times 100 (12). All clearance values were considered, including those within normal limits at baseline, since the trend over time could provide information on graft function (12). The PIM-2 pediatric mortality index was calculated for all patients within 24 h of admission to intensive care.

According to institutional protocol, red blood cell transfusion was ordered when hemoglobin levels were below 4.3 mmol/L (7 gr/dL), and plasma was ordered when there was active bleeding through the drains, with hemodynamic instability.

The patients included in the liver transplant list were determined according to the King's College criteria. Cold ischemia time was defined as the time from donor vessel clamping and cold preservation solution flushing to graft insertion in the recipient's abdominal cavity (13). The anhepatic phase was defined as the time from physical extraction of the recipient's liver until graft recirculation (14).

### Statistical Analysis

Qualitative clinical and demographic variables are reported as absolute and relative frequencies and were compared according to the type of donor (living/cadaveric) using the Chi-square independence test. Quantitative variables are reported as median and interquartile range and were compared between groups using the Mann-Whitney U test. To determine the best and most accurate cut-off point for lactate, a receiver operating characteristic (ROC) analysis was performed. This approach was used since the ROC curve is not dependent on the prevalence of the disease, but is dependent on the patient's characteristics and the disease spectrum, which may behave differently in children after liver transplantation.

To model the evolution of lactate concentration (LC) over time, we proposed a mixed log-linear model in which we considered the correlation of repeated LC measurements at different times in a single patient by means of a random effect. The model considered the patient's initial LC and estimated its degradation rate over time. Through this approach, we established groups of patients with similar lactate disintegration parameters and determined their clinical characterization through a decision tree structure. Further details of the model are described in the Supplementary File 1.

In this context, we will use the approach proposed by Fokkema et al. in 2018, consisting of a classification and regression model based on generalized mixed linear models (15). This approach allows us to find groups of patients with similar lactate disintegration parameters, and the clinical characterization of these groups is established through a decision tree structure. Finally, the main procedure-related complications (such as arterial or portal thrombosis, biliary complications and death) are presented using absolute and relative values and are compared according to type of transplant using the Chi-square test. All statistical analyses were done using R software version 4.1.0, and the significance level was set to 5%.

## Results

During the study period, 168 liver transplants were performed on children. Of these, 23 patients were excluded for the reasons presented in Supplementary File 2. A total of 145 patients were included in this study. The population characteristics are described in Table 1.

**Table 1:** Distribution of clinical variables in the study population according to type of donor.

**Variable**	**Total (*n* = 145)**	**Living donor (*n* = 107)**	**Cadaveric donor (*n* = 38)**	***P*-value**
Sex. Female *n* (%)	86 (59.3)	65 (60.7)	21 (55.3)	0.554
Age (months). median (IQR)	14 (8–60)	11 (7–30)	17 (8–56)	<0.001
Weight at transplant (kg). median (IQR)	8 (6–18)	7 (6–12)	25 (10–39)	<0.001
**Type of graft**. ***n*** **(%)**
Partial	123 (84.8)	105 (98.1)	18 (47.4)	<0.001
Complete	16 (11.0)	2 (1.9)	14 (36.8)	<0.001
Split	6 (4.1)	–	6 (15.8)	–
Hyper-reduced liver. *n* (%)	9 (6.2)	9 (8.4)	–	1.000
**Primary diagnosis**. ***n*** **(%)**
Biliary atresia	82 (56.5)	69 (64.5)	13 (34.2)	<0.001
Malignant neoplasm	13 (8.9)	11 (10.3)	2 (5.2)	0.571
Acute hepatic necrosis	11 (7.5)	4 (3.7)	7 (18.4)	0.014
Cholestatic cirrhosis	10 (6.8)	7 (6.5)	3 (7.9)	1.000
Metabolic disease	9 (6.2)	5 (4.7)	4 (10.5)	0.365
Non-cholestatic cirrhosis	7 (4.8)	4 (3.7)	3 (7.9)	0.531
Other	13 (8.9)	7 (6.5)	6 (15.8)	0.632
PIM2 score. median (IQR)	7.0 (4.8–11.1)	7.2 (5.1–11.9)	6.4 (4.1–9.8)	0.138
Vasoactive score. median (IQR)	15 (6.6–42.2)	12.5 (6.0–33.5)	34 (11–67)	0.003
Length of anhepatic phase (minutes). median (IQR)	55 (47–65)	54 (47–65)	60 (48–68)	0.201
Cold ischemia time (minutes). median (IQR)	335 (260–400)	292 (245–350)	480 (407–620)	<0.001
Fluids administered during surgery (mL/kg). median (IQR)	43.4 (24.6–70.1)	40.8 (20.6–68.2)	45.1 (25.3–71.7)	<0.001
Lactate on PICU admission (mmol/L). median (IQR)	2.4 (1.5–4.2)	2.2 (1.5–3.9)	2.7 (1.6–5.7)	0.169
Length of stay in PICU (days). median (IQR)	7 (4–10)	6 (4–10)	7 (4–13)	0.555
Days of mechanical ventilation. median (IQR)	2 (1–4)	2 (1–6)	2 (1–3)	0.817

*PIM-2, Pediatric index mortalitly-2. Vasoactive-inotropic score (VIS) calculated as follows: dopamine (mcg/kg/min) + dobutamine (mcg/kg/min) + 100 × epinephrine (mcg/kg/min) + 10 × milrinone (mcg/kg/min) + 10,000 × vasopressin (U/kg/min) + 100 × norepinephrine (mcg/kg/min)*.

The median procedure time was 380 min (IQR 326–450). The PIM-2 was not correlated with any lactate level during the post-operative period. The length of stay in the intensive care unit was correlated with the serum lactate concentration only at T4 (Rho = 0.04; *p* = 0.5). The most frequently used blood products were red blood cells (55.8%), fresh frozen plasma (21.2%) and platelet concentrate (11.5%), as well as cryoprecipitates (11.5%). Altogether, 80.6% (*n* = 117) of the patients required vasoactive medications and children who received a cadaveric transplant had a higher score and required more post-operative support (*p* = 0.003).

A total of 17.2% (*n* = 25) of the patients had post-operative surgical complications. Of these, 36% (*n* = 9) required biliary reconstruction, 24% (*n* = 6) had hemoperitoneum, 16% (*n* = 4) had a biliary fistula, 8% (*n* = 2) required exploratory laparotomy, 8% (*n* = 2) had intestinal perforation, 4% (*n* = 1) had chylous ascites, and 4% (*n* = 1) required peritoneal lavage (Table 2). Altogether, 3.4% (*n* = 5) of the study patients had biliary complications, with the most frequent being biliary fistula in 80% (*n* = 4) and bile collection in 20% (*n* = 1), all cases corresponding to children receiving living-donor transplants. The main causes of death were septic shock (53.8%), distributive shock (15.3%), hemorrhagic shock (15.3%) and two cases of death from bleeding, one in the central nervous system and the other due to acquired hemophilia. The most frequent complications are shown in Table 2.

**Table 2:** Distribution of clinical outcomes in the study population according to type of donor.

**Variable**	**Total (*n* = 145)**	**Living donor (*n* = 107)**	**Cadaveric donor (*n* = 38)**	***P*-value**
Surgical complications *n* (%)	25 (17.2)	19 (18)	6 (15.7)	0.102
Graft complications *n* (%)	6 (4.1)	2 (1.9)	4 (10.5)	<0.001
Arterial or venous thrombosis *n* (%)	9 (6.2)	4 (3.7)	5 (13.2)	0.417
Death *n* (%)	12 (8.3)	6 (5.6)	6 (15.7)	0.009

**Figure 1:**
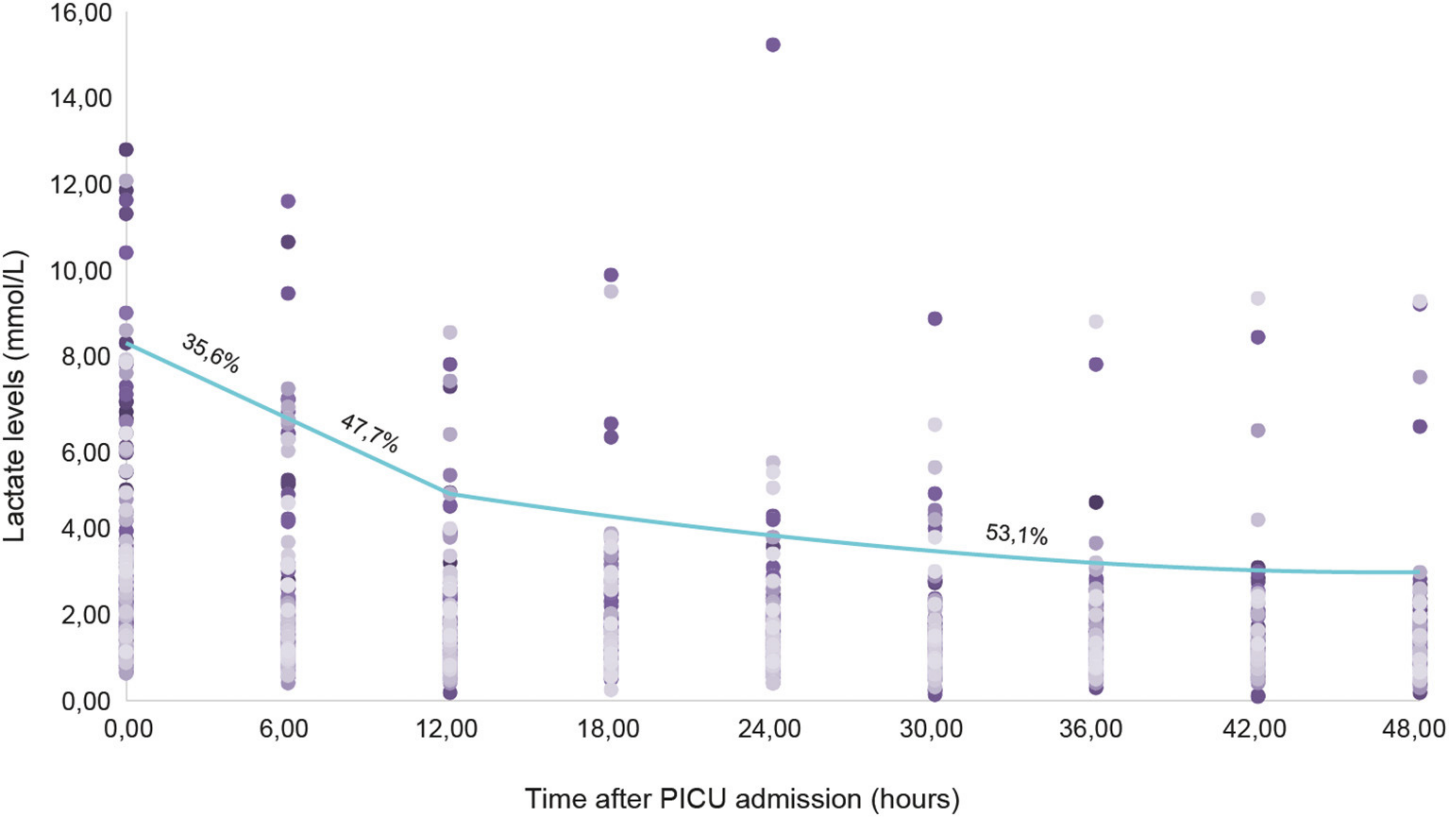
Clearance of lactate levels in the first 48 post-operative hours.

The median lactate at T0 was 2.41 mmol/L (IQR 1.5–4.2); at T1 it was 1.55 mmol/L (IQR 1.0–2.5), at T2 it was 1.26 mmol/L (IQR 1.0–1.8), and at T3 it was 1.13 mmol/L (IQR 0.8–1.6). Clearance at the first 6 h was 35.6% in all patients; it was 47.7% at 24 h, and 53.1% at 48 h (Figure 1). The number of patients with abnormal lactate levels at T0 was 89 (61%); at T1 it was 54 (37%) and after T2 it was 17 (11%).

**Figure 2:**
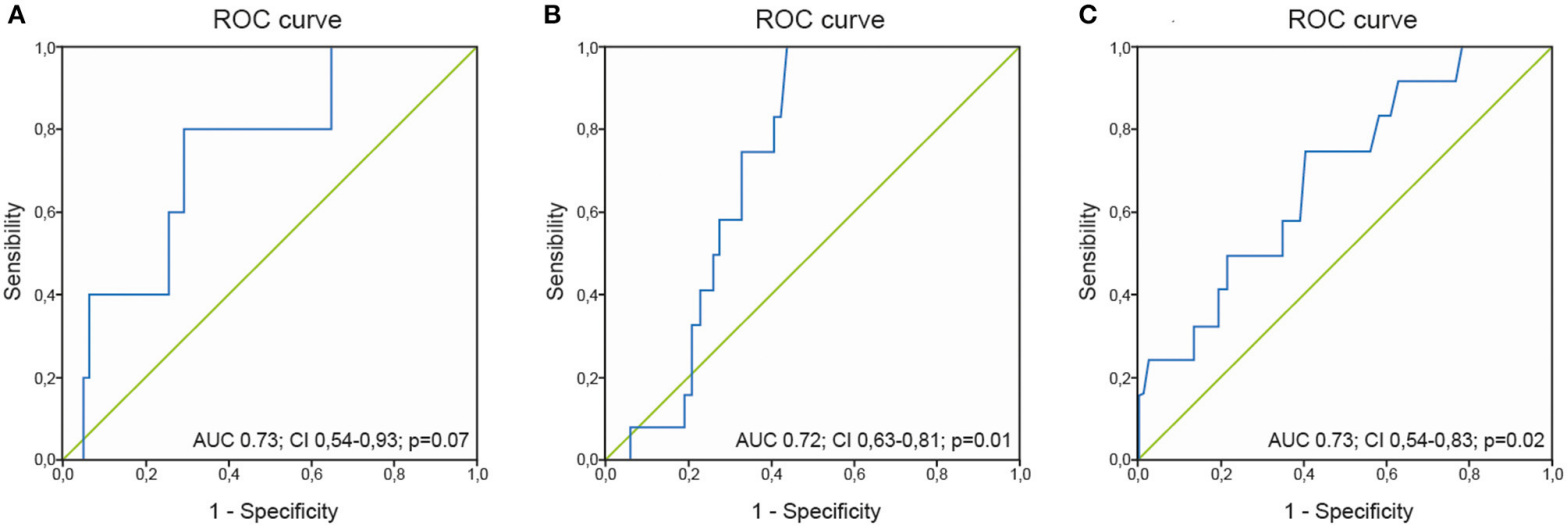
ROC curve for lactate levels at admission and biliary complications **(A)** or mortality **(B)**, and ROC curve for lactate levels at 6 h and mortality **(C)**.

We found that a serum lactate level > 3.0 mmol/L on PICU admission had an 80% sensitivity and 65% specificity (AUC 0.73; CI 0.54–0.93, *p* = 0.05) for predicting biliary complications, venous or arterial thrombosis (62% sensitivity and 64% specificity; AUC 0.58; CI 0.40–0.77; *p* = 0.4), and 90-day mortality (76% sensitivity and 65% specificity, AUC 0.72; CI 0.63–0.81; *p* = 0.01). In this regard, if elevated lactate persists and is >2 mmol/L 6 h after PICU admission, it continues to be useful for predicting mortality (75% sensitivity and 60% specificity; AUC 0.70; CI 0.54–0.83; *p* = 0.02) (Figure 2).

Total procedure time had a slight, non-significant correlation with lactate levels at T0 (Rho = 0.12; *p* = 0.16), T1 (Rho = 0.12; *p* = 0.16) and T2 (Rho = 0.11; *p* = 0.18), but was not correlated with T3 (Rho = −0.05; *p* = 0.5). Cold ischemia time was weakly correlated with all the measurement times (T0 Rho = 0.12, *p* = 0.13; T1 Rho = 0.01, *p* = 0.03; T2 Rho = 0.01, *p* = 0.87; T3 Rho = 0.03, *p* = 0.71), as was the length of the anhepatic phase (T0 *r* = 0.16, *p* = 0.05; T1 *r* = 0.11, *p* = 0.23; T2 *r* = 0.22, *p* < 0.01; T3 *r* = 0.09, *p* = 91), with no statistical significance found.

**Figure 3:**
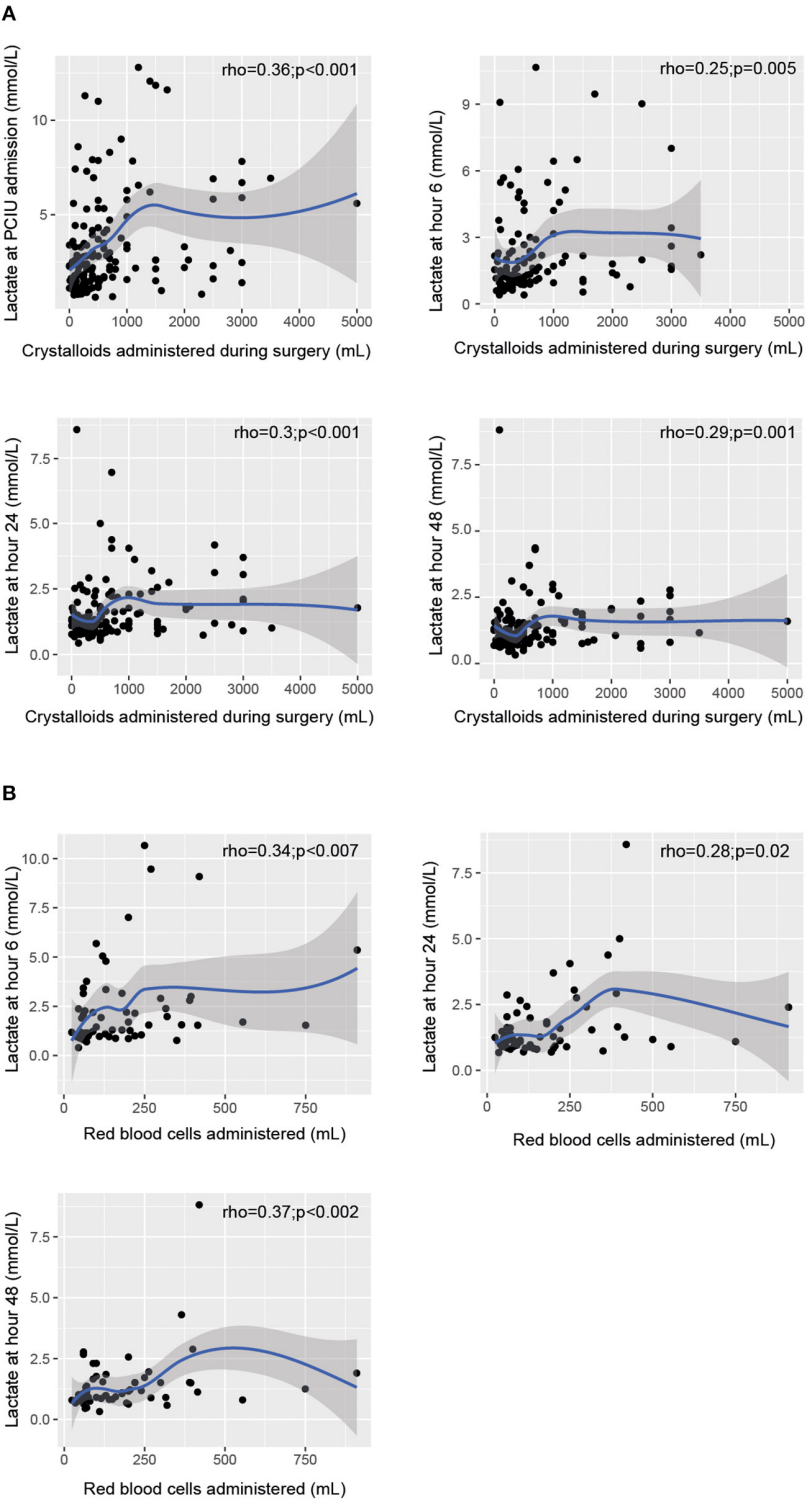
Relationship between serum lactate levels at the various assessment times and the volume of fluids administered during surgery **(A)**. Relationship between post-operative lactate levels and the volume of red blood cells administered **(B)**.

The median volume of fluids administered during surgery was 43.4 mL/kg (IQR 24.6–70.1). Serum lactate levels at the four measurement times had a slight to moderate inverse correlation with the volume of fluids administered during surgery (Figure 3A). Likewise, an inverse relationship was found between serum lactate levels at T1, T2, and T3 and the volume of red blood cells administered post-operatively (Figure 3B). With regard to the volume of plasma transfused post-operatively, we found a slight correlation with T3 (Rho = 0.33; *p* = 0.12) and a moderate one with T1 (Rho = 0.51; *p* = 0.02), T2 (Rho = 0.45; *p* = 0.04) and T4 (Rho = 0.55; *p* < 0.01).

Lactate levels were significantly correlated with the VIS after the first post-operative hour (T1 *r* = 0.39, *p* = < 0.001; T2 *r* = 0.45, *p* < 0.01; T3 *r* = 0.48, *p* < 0.01).

**Figure 4:**
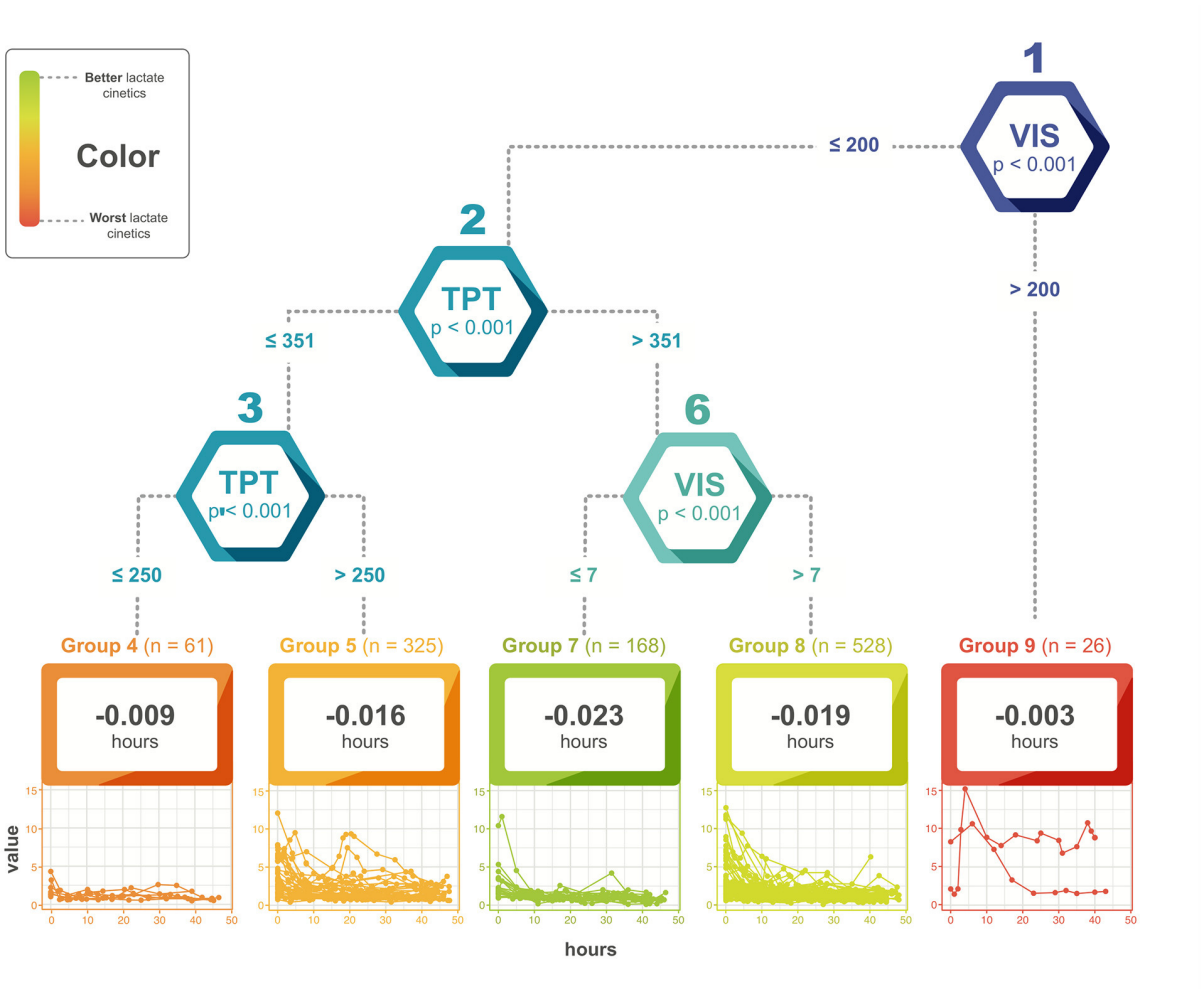
Relationship between vasoactive-inotropic score, total procedure time and lactate levels. VIS, vasoactive-inotropic score; TPT, total procedure time.

Similarly, for total procedure time > 350 min, a VIS > 7 was found to be related to elevated lactate levels and worse outcomes (*p* < 0.001) (Figure 4). Likewise, higher lactate levels and lack of good clearance were related to the presence of *tardus et parvus* waveforms on hepatic Doppler (*p* = 0.001) and graft complications such as primary dysfunction (*p* < 0.001) and arterial thrombosis (*p* < 0.001), as well as neurological complications (*p* = 0.04). The presence of surgical complications and post-operative infections was also related to higher levels of lactate at T2 (*p* < 0.001).

**Figure 5:**
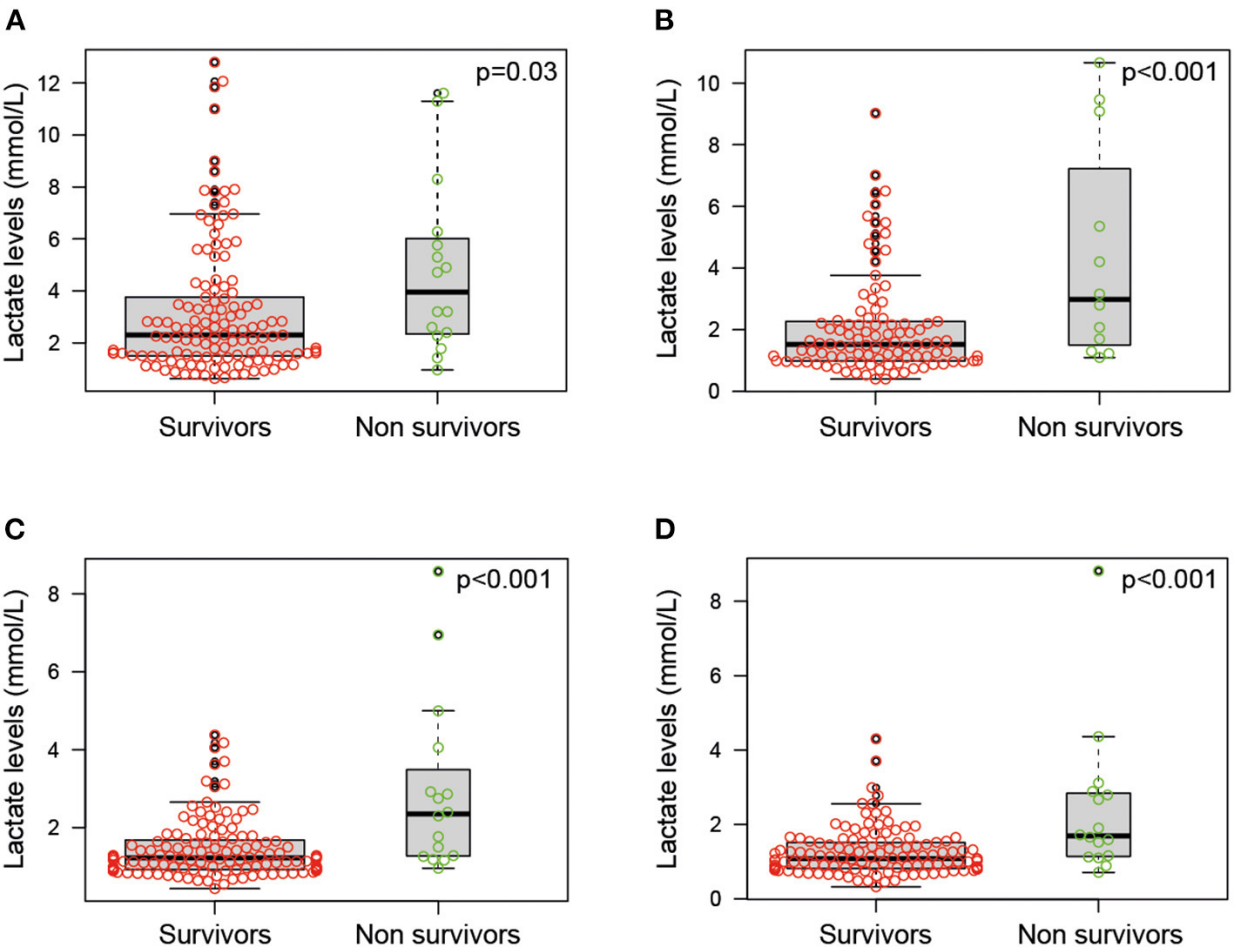
Lactate levels in patients who did not survive 90 days after surgery **(A)** during the first hour after admission, **(B)** between 1 and 6 h after admission, **(C)** between 6 and 24 h after admission, and **(D)** between 24 and 48 h after admission.

At all times, the highest lactate values were recorded in patients with a pre-transplant diagnosis of acute liver cirrhosis (mean 5.18 ± 2) and malignant neoplasms (mean 7.06 ± 3.1) (*p* = 0.01). Higher lactate levels were directly correlated at all assessment times with patients who did not survive 90 days after surgery (Figure 5).

## Discussion

In this study, we found that a serum lactate concentration > 3.0 mmol/L on admission to intensive care, or a value persistently > 2 mmol/L 6 h after surgery, was associated with greater mortality in children following liver transplantation. We also found that high concentrations were associated with the presence of alterations on liver Doppler, primary dysfunction and arterial thrombosis. In the same vein, the lactate level on admission was found to be correlated with the volume of fluids administered during surgery. In addition, a surgery lasting longer than 350 min, together with the need for high vasopressor support, was associated with the presence of more surgical and medical post-operative complications.

The SCCM consensus recommends using lactate as an indirect marker of tissue perfusion and reports some studies which have related it to adverse outcomes and death in septic shock (16, 17). Jaiswal et al. reported that a lactate level > 2.5 mmol/L 6 h after admission was a good predictor of mortality (85% sensitivity and 74% specificity) in pediatric patients with a diagnosis of sepsis and in other critical care patients (18–22).

The behavior of serum lactate has been particularly studied following liver transplantation in adults. Murphy et al. found that portal lactate increases significantly compared to pre-transplant levels, without overall changes in the splanchnic gradient. These findings suggest the existence of intestinal lactate production, which does not ultimately affect systemic levels if the graft functions adequately (23). De Gasperi et al. described the behavior of lactate in the first 48 h in patients with adequate graft function, finding that in the first 12 h the levels were lower than 4 mmol/L, and in the following 24 and 48 h were lower than 2 mmol/L (24). Those with inadequate function had levels close to 4 mmol/L at 24 and 48 h. In our study, we found that after being admitted to intensive care, the persistence of lactate above 2 mmol/L was associated with similar outcomes, especially graft dysfunction.

Serum lactate levels depend on the balance between production and elimination (9–11). Approximately 60% of the lactate produced is metabolized in the liver. It could rise in patients with liver failure, as well as in the different phases of liver transplantation (11, 12). Specifically, lactate elevation would be expected in the anhepatic phase of surgery, with its level directly related to the duration of this phase (13, 14, 25, 26). Later, in the reperfusion phase, with adequate functioning of the liver graft, adequate clearance should begin until normal levels are reached (14). Therefore, an inadequate decrease in its value could be associated with graft malfunction.

Various measurements of lactate as a biomarker have been proposed for study in relation to post-operative liver transplant outcomes. Some authors propose a clearance percentage cut-off in the first hours of monitoring. In adults, Kim et al. found a clearance cut-off of 25% in the first 6 h to be useful for detecting allograft rejection (76% sensitivity and 77% specificity, AUC 0.82; *p* < 0.001) (10) while for Wu et al., this same time point cut-off was associated with greater inpatient mortality and poor allograft response (95% sensitivity, 88% specificity; AUC 0.961 ± 0.01; *p* < 0.001) (12). Whether the wide time range from T1 to T2 and T2 to T3 could influence lactate level variability is unknown, although the trend observed across these time periods is for it to remain stable or decrease.

On the other hand, if a cut-off point of absolute post-operative lactate levels is considered, Takahashi et al. reported a cut-off point of more than 4.45 mmol/L immediately after reperfusion and 3.45 mmol/L in the immediate post-operative period, along with a clearance of 0.2 mmol/L per hour (AUC 0.63; 0.68–0.60) for early graft dysfunction in adults (12). In our study, a cut-off point of more than 3 mmol/L in the immediate post-operative period (first hour after admission to the PICU) was related to biliary complications, vascular thrombosis and death. In adult patients, Jipa et al. found a similar cut-off point to what was found in our study (>3 mmol/L), which was correlated with intraoperative blood loss when evaluated in the immediate post-operative period (27, 28).

In our study, the highest lactate levels were directly correlated with the total procedure time. Takahashi et al. found that a warm ischemia time ≥41 min was the only factor associated with delayed lactate clearance (12). We did not find a significant correlation between post-operative lactate levels and the specific length of the various surgical times. However, we did find an inverse relationship between the fluids administered during surgery, the need for red blood cell or plasma transfusions and serum lactate levels. Takahashi et al. found no correlation between the volume of fluids administered during surgery and graft function (12). We believe that as long as a good tissue perfusion pressure is maintained, with a homogenous microcirculation flow, tissue oxygenation can be maintained within adequate limits to allow good lactate clearance and lower post-operative levels.

We consider that our study has several limitations. The results are obtained from the experience of a single healthcare center. We did not evaluate whether the unsatisfactory outcomes were similar at different points in the center's experience. In addition, serum lactate levels may be influenced by the presence of citrate or other preservatives in the blood product transfusions, especially red blood cells. This could have affected the lactate levels, which were significantly higher in the group with the most transfusions. Likewise, serum lactate has some limitations as a biomarker. Its value may be influenced by beta-adrenergic activity, and adrenaline is a commonly used inotrope in pediatrics. This could alter the observed values and even the clearance rate. This could be a bias in our study since there was a wide range of time at T2 prior to the next measurement. This is why trends were evaluated and compared with the outcomes. In fact, lactate is a non-specific biomarker and would have a smaller area under the curve to discriminate mortality with respect to scales such as PIM-2 (29). The statistical model used a mathematical model to better treat the continuous variable that could not be adapted to multiple potential confounding factors (cold ischemia time, length of anhepatic phase, patient's weight, transfusions, or the PIM2) given the non-specificity of lactate as a perfusion biomarker. However, persistently elevated lactate was associated with an unsatisfactory clinical or surgical evolution. Another aspect to consider is the change in management protocols over time and the new technologies introduced during the study period, which could have affected patient outcomes in the latter years compared to the initial ones.

## Conclusions

In children recovering from liver transplantation, serum lactate levels are associated with post-operative surgical complications and mortality, although we were not able to identify if this relationship was independent from multiple other confounders given that lactate is a non-specific biomarker. We found that high levels are associated with more alterations on the post-operative liver Doppler, primary dysfunction and arterial thrombosis. Interestingly, there is an inverse correlation between the volume of fluids administered during surgery and serum lactate levels. When the procedure is prolonged and high vasopressor support is required, lactate levels are higher and are associated with a higher frequency of complications.

## Data Availability Statement

The original contributions presented in the study are included in the article/Supplementary Material, further inquiries can be directed to the corresponding author/s.

## Ethics Statement

The studies involving human participants were reviewed and approved by the Ethics Committee of the Fundación Cardioinfantil-Instituto de Cardiología (committee reference number CEIC-3996-2019). Written informed consent to participate in this study was provided by the participants' legal guardian/next of kin.

## Author Contributions

JF-S, MW-C, RH-S, HM, KM-M, NM, JD, LA, CS, and JR contributed to designing and performing the study. JF-S, MW-C, and RH-S participated in data collection. JF-S supervised study development and data collection. All authors contributed to drafting the manuscript, reviewing the final article, approved the final manuscript as submitted and agree to be accountable for all aspects of the work.

## Conflict of Interest

The authors declare that the research was conducted in the absence of any commercial or financial relationships that could be construed as a potential conflict of interest.

## Publisher's Note

All claims expressed in this article are solely those of the authors and do not necessarily represent those of their affiliated organizations, or those of the publisher, the editors and the reviewers. Any product that may be evaluated in this article, or claim that may be made by its manufacturer, is not guaranteed or endorsed by the publisher.

## Supplementary Material

Supplementary File 1 Model of the evolution of lactate concentration.

Supplementary File 2 Flowchart of study patient selection.
