# Supplementary material for: Association Between Serum Lactate and Unsatisfactory Outcomes in Critically Ill Children in the Immediate Post-operative Period of Liver Transplantation
Source: Front Pediatr. 2022 Jan 24;9:796504. doi: 10.3389/fped.2021.796504 (PMC8818884; doi:10.3389/fped.2021.796504)
Supplement: Supplementary File 1 — Model of the evolution of lactate concentration. [file Data_Sheet_1.docx]

**Supplementary file 1**

**Model of the evolution of lactate concentration**

To model the evolution of lactate concentration (LC) over time, we proposed a mixed log-linear model in which we consider the correlation of repeated LC measurements at different times in a single patient by means of a random effect. The deterministic component of the model is as follows: $Ln\left( {LC}_{t_{i}} \right)= \beta_{0}+\beta_{1}t_{i}$; where $Ln$ is the natural logarithmic function, and ${LC}_{t_{i}}$ is the LC measurement at time $t$ for the “i”th patient ($i$). This model is closely related to the disintegration models used in various fields such as nuclear physics, or the Michaelis-Menten enzyme kinetics equations. The general formula establishes that the degradation of the concentration of a substance (such as an isotope or an enzyme substrate) over time depends on the initial quantity of the substance, establishing the following differential equation system: $dN=\lambda Ndt$, where $dN$ is the change in the concentration of the substance and $dt$ is the change over time. The classical solution for this differential equation is the following: $N\left( t \right)=N(t_{0})e^{-\lambda t}$, where $N\left( t \right)$ is the concentration of the substance at time $t$, $N(t_{0})$ is the initial concentration of the substance, and $-\lambda$ is a parameter related to the rate of disintegration of the given substance. This equation allows linearization as follows: $Ln\left( N\left( t \right) \right)=Ln\left( N\left( t_{0} \right) \right)-\lambda t$. Thus, in the formula we used to model LC over time, $\beta_{0}$ would be related to the patient’s initial lactate concentration and the parameter $\beta_{1}$ would quantify the magnitude of its degradation rate.

In this context, we will use the approach proposed by Fokkema et al. in 2018, consisting of a classification and regression model based on generalized mixed linear models (15). This approach allows us to find groups of patients with similar lactate disintegration parameters, and the clinical characterization of these groups is established through a decision tree structure.
