# Supplementary figures and images for: Association Between Serum Lactate and Unsatisfactory Outcomes in Critically Ill Children in the Immediate Post-operative Period of Liver Transplantation
Source: Front Pediatr. 2022 Jan 24;9:796504. doi: 10.3389/fped.2021.796504 (PMC8818884; doi:10.3389/fped.2021.796504)

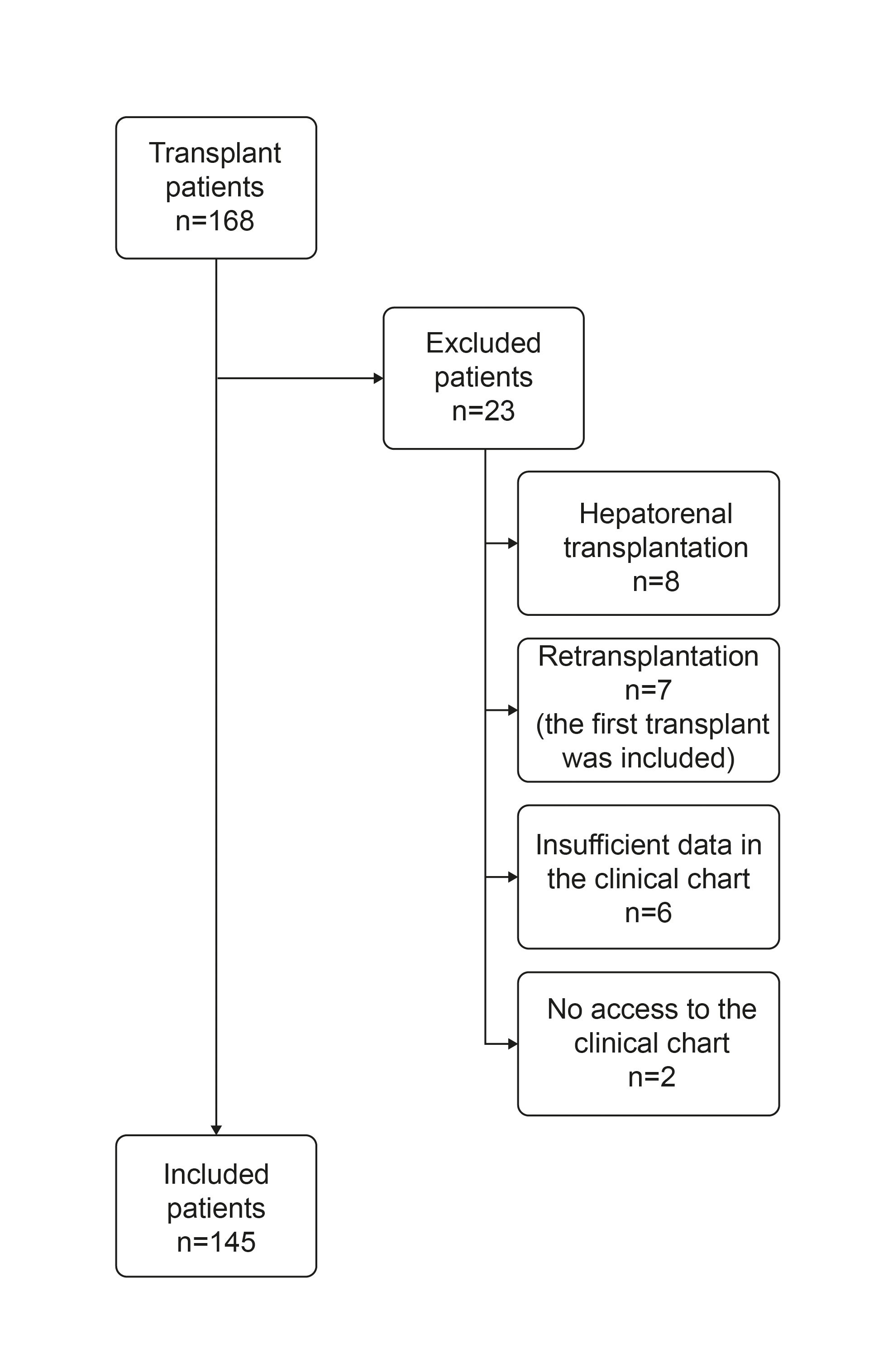

Supplement: Supplementary File 2 — Flowchart of study patient selection. [file Image_1.TIF]
